# Reporting of methodologies used for clonogenic assays to determine radiosensitivity

**DOI:** 10.1093/jrr/rraa064

**Published:** 2020-08-22

**Authors:** Takahiro Oike, Shuichiro Komatsu, Yuka Komatsu, Ankita Nachankar, Narisa Dewi Maulany Darwis, Atsushi Shibata, Tatsuya Ohno

**Affiliations:** Department of Radiation Oncology, Gunma University Graduate School of Medicine, 3-39-22, Showa-machi, Maebashi, Gunma 371-8511, Japan; Gunma University Heavy Ion Medical Center, 3-39-22, Showa-machi, Maebashi, Gunma 371-8511, Japan; Department of Radiation Oncology, Gunma University Graduate School of Medicine, 3-39-22, Showa-machi, Maebashi, Gunma 371-8511, Japan; Department of Radiation Oncology, Gunma University Graduate School of Medicine, 3-39-22, Showa-machi, Maebashi, Gunma 371-8511, Japan; Department of Radiation Oncology, Gunma University Graduate School of Medicine, 3-39-22, Showa-machi, Maebashi, Gunma 371-8511, Japan; Department of Radiation Oncology, Gunma University Graduate School of Medicine, 3-39-22, Showa-machi, Maebashi, Gunma 371-8511, Japan; Department of Radiation Oncology, Faculty of Medicine Universitas Indonesia – Dr. Cipto Mangunkusumo Hospital, Jl. P. Diponegoro no. 71, Jakarta 10430, Indonesia; Gunma University Initiative for Advanced Research (GIAR), 3-39-22, Showa-machi, Maebashi, Gunma 371-8511, Japan; Department of Radiation Oncology, Gunma University Graduate School of Medicine, 3-39-22, Showa-machi, Maebashi, Gunma 371-8511, Japan; Gunma University Heavy Ion Medical Center, 3-39-22, Showa-machi, Maebashi, Gunma 371-8511, Japan

**Keywords:** clonogenic assays, cancer, radiation, radiosensitivity, replicates

## Abstract

Radiotherapy treatment strategies should be personalized based on the radiosensitivity of individual tumors. Clonogenic assays are the gold standard method for *in vitro* assessment of radiosensitivity. Reproducibility is the critical factor for scientific rigor; however, this is reduced by insufficient reporting of methodologies. In reality, the reporting standards of methodologies pertaining to clonogenic assays remain unclear. To address this, we performed a literature search and qualitative analysis of the reporting of methodologies pertaining to clonogenic assays. A comprehensive literature review identified 1672 papers that report the radiosensitivity of human cancer cells based on clonogenic assays. From the identified papers, important experimental parameters (i.e. number of biological replicates, technical replicates, radiation source and dose rate) were recorded and analyzed. We found that, among the studies, (i) 30.5% did not report biological or technical replicates; (ii) 47.0% did not use biological or technical replicates; (iii) 3.8% did not report the radiation source; and (iv) 32.3% did not report the dose rate. These data suggest that reporting of methodologies pertaining to clonogenic assays in a considerable number of previously published studies is insufficient, thereby threatening reproducibility. This highlights the need to raise awareness of standardization of the methodologies used to conduct clonogenic assays.

## INTRODUCTION

Radiotherapy, along with surgery and chemotherapy, is one of the major pillars of cancer treatment. In the clinic, the dose required to eradicate a tumor varies widely, even among tumors that arise from the same organs [1]. This indicates the need for a personalized radiotherapy strategy based on the radiosensitivity of individual tumors. Clonogenic assays are the gold standard method for assessing radiosensitivity *in vitro* [2]. Historic data suggest that the radiosensitivity of cancer cells measured in clonogenic assays is associated with the clinical response of a tumor to radiotherapy [3–6]. More recently, we and others demonstrated that radiotherapy outcomes are associated with the genetic profile of the tumor (e.g. *EGFR* mutations) identified by correlation analysis of clonogenic assay- and omics-data [7–10]. These data indicate that inter-study comparison and integration of clonogenic assay data reported in the literature have huge potential with respect to developing precision medicine approaches in the field of radiotherapy [11].

In scientific research, reproducibility is the critical factor that enables comparison of experimental results from different studies [12]. Reproducing prior results is difficult if the reporting of methodologies in the original study is insufficient [12]. In recent years, attempts have been made to improve the reporting of methodologies in scientific publications, e.g. the FAIR (findable, accessible, interoperable and reusable) principles [13]. However, the reality is that the reporting standards of methodologies pertaining to clonogenic assays remain unclear. The lack of evidence makes it difficult to validly compare published clonogenic assay data. To address this, the present study aimed to examine the reporting of clonogenic assay methods in the literature. We conducted a comprehensive literature search and identified 1672 papers reporting the use of clonogenic assays to determine radiosensitivity. We then conducted qualitative analysis of the reporting of important experimental parameters pertaining to clonogenic assays.

**Fig. 1.**
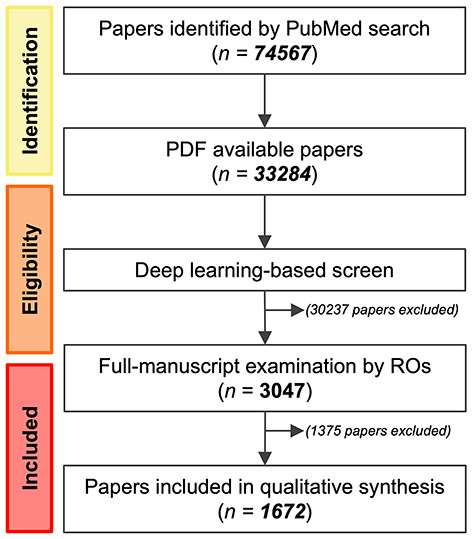
Schematic showing the flow of the literature review. RO = radiation oncologist.

## MATERIALS AND METHODS

### Literature screen

Figure 1 shows the scheme used for the literature review. A PubMed search was performed on 29 December 2018 for each of 1039 human cancer cell lines registered to the Cancer Cell Line Encyclopedia (CCLE) [14] using the terms ‘*cell line name* AND (X-rays OR gamma rays OR radiation)’. The search identified 74 567 papers. Among them, a PDF file was available for 33 284. All available PDF files were subjected to a deep learning-based screen that identifies the papers containing radiosensitivity data obtained using clonogenic assays [15]. The program used for the deep leaning-based screen consists of three classifiers. Classifier #1 identifies publications that contain semi-logarithmic graphs showing radiosensitivity data derived from clonogenic assays by using two deep convolutional neural network architectures, i.e. faster regions convolutional neural networks with inception resnet v2 (fRCNN-IRv2) and VGG-16, and a text mining module, optical character recognition (OCR). Classifier #2 identifies publications that contain bar graphs showing radiosensitivity data derived from clonogenic assays by using fRCNN-IRv2 and OCR. Classifier #3 identifies publications that contain keywords related to radiosensitivity data as assessed by clonogenic assays by using a text-mining technology. The logical sum of the results generated by the three classifiers is exported as the outcome of the screen program. The screen identified 3047 papers. Radiation oncologists (S.K., Y.K., A.N. and N.D.M.D.) examined all 3047 papers in their entirety based on the following eligibility criteria. Inclusion criteria: a paper written in English that reports radiosensitivity (at least that for 2 Gy) of the cell line of interest determined using clonogenic assays. Exclusion criteria: concomitant use of reagents, plasmids or small interfering RNAs (siRNAs) alongside radiation (treatment with solvent, scramble-siRNA or empty-vector as a control was accepted) or the use of particle radiation. The eligibility of one paper was double-checked by independent radiation oncologists.

### Data acquisition

From the papers that met the eligibility criteria, the number of experiments (*n*_E_), the number of samples per experiment (*n*_S_), radiation source and dose rate used for clonogenic assays were recorded by radiation oncologists (S.K., Y.K., A.N. and N.D.M.D.). The records were double-checked by independent radiation oncologists. If a paper reported multiple values for *n*_E_ or *n*_S_, then the minimum value was recorded, whereas the mean value was recorded for the dose rate.

## RESULTS

The comprehensive literature review focused on the 1039 human cancer cell lines registered in the CCLE [14]. From 74 567 candidate papers, a deep learning-based screen [15] followed by full-manuscript examination identified 1672 papers that report the use of clonogenic assays to determine the radiosensitivity of these cell lines (Fig. 1, Supplementary Data 1, see online supplementary material). The details of the literature review are described in the Materials and methods section.

**Fig. 2.**
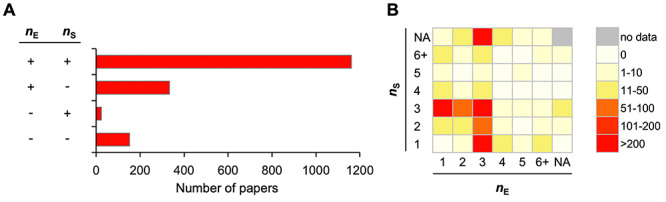
Biological and technical replicates for clonogenic assays reported in the literature (*n* = 1672). *n*_E =_ Number of experiments; *n*_S =_ number of samples per experiment. (**A**) Number of papers stratified according to the presence or absence of reporting the *n*_E_ and *n*_S_. (**B**) Heatmap showing the number of papers stratified according to the reported *n*_E_ and *n*_S_. NA, not assessable.

In clonogenic assays designed to test *in vitro* radiosensitivity, cultured cells are prepared on plates (or in suspensions), irradiated, and then incubated for an additional 10–14 days; next, the colonies are stained and counted (Table 1). In this procedure, the cell culture conditions pre- and post-irradiation are a source of biological variance; this is mitigated by repeating the entire experiment (i.e. biological replicates) [16, 17]. Meanwhile, preparation and irradiation of cells and staining and counting of colonies are a source of technical variance; this is mitigated by increasing the number of samples in an experiment (i.e. technical replicates) [16, 17]. Therefore, we analyzed papers to assess reporting of the number of experiments (i.e. *n*_E_) and the number of samples per experiment (i.e. *n*_S_). Of the 1672 papers identified, 1162 (69.5%) reported both *n*_E_ and *n*_S_; 334 (20.0%) reported *n*_E_ only; 24 (1.4%) reported *n*_S_ only; and 152 (9.1%) reported neither *n*_E_ nor *n*_S_ (Fig. 2A). Of the 1162 papers that reported both *n*_E_ and *n*_S_, the common combinations of *n*_E_ and *n*_S_ were 1–3, 3–1, 3–3, 3–2 and 2–3 [in 356 (30.7%), 251 (21.6%), 235 (20.2%), 59 (5.1%) and 53 (4.5%) papers, respectively] (Fig. 2B). Other *n*_E_–*n*_S_ combinations were observed in < 3% of papers. Of the 334 papers that reported *n*_E_ only, 278 (83.2%) reported an *n*_E_ of 3. Of the 24 papers that reported *n*_S_ only, 15 (62.5%) reported an *n*_S_ of 3.

**Table 1:** Experimental procedure and type of variance in clonogenic assays

Experimental procedure	Type of variance
Cell culture	Biological
Cell preparation	Technical
Irradiation	Technical
Incubation	Biological
Colony staining	Technical
Colony count	Technical

**Fig. 3.**
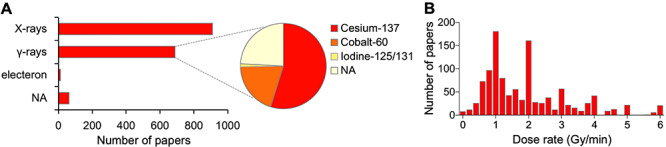
Irradiation setting for clonogenic assays reported in the literature. (**A**) Radiation source (*n* = 1672). NA, not assessable. (**B**) Dose rate (*n* = 1132). Bin size of the histogram, 0.2. Fourteen data points (1.2%) were out of the range of the *x*-axis.

In addition to biological and technical replicates, we analyzed reporting of the radiation source and dose rate; this is because these factors possibly affect the assay results even though an identical dose was used among assays [2]. Of the identified 1672 papers, 910 (54.4%), 688 (41.1%) and 12 (0.7%) reported the use of X-rays, γ-rays and electrons, respectively, whereas 62 (3.8%) did not report the radiation source (Fig. 3A). The source of γ-rays was ^137^Cs, ^60^Co and ^125^I or ^131^I [in 377 (54.8%), 137 (19.9%) and eight (1.1%) papers, respectively]; however, 166 papers (24.1%) did not report the source of γ-rays (Fig. 3A). The dose rate was reported in 1132 of the 1672 papers (67.7%). The reported dose rate ranged from 0.002 to 214 Gy/min, an average ± standard deviation of 2.2 ± 6.5 Gy/min (Fig. 3B).

## DISCUSSION

To the best of our knowledge, this is the largest study to date that has investigated the reporting of clonogenic assay methods. The main findings are: (i) 30.5% of studies did not report biological or technical replicates; (ii) 47.0% did not employ biological or technical replicates; (iii) 3.8% did not report the radiation source; and (iv) 32.3% did not report the dose rate. These data suggest that reporting of methodologies pertaining to clonogenic assays in a considerable number of previously published studies is insufficient, thereby threatening reproducibility. The data presented herein will raise awareness about the importance of detailed and consistent reporting of the methods used to conduct clonogenic assays.

The number of replicates employed in an assay varies according to the type of experiment and is often affected by factors unrelated to statistics, e.g. historic and economic contexts. For example, the field of genome sequencing does not demand replication owing to the high cost per assay [16]. Clonogenic assays are time-consuming, i.e. they take 10–14 days to complete. This may make one hesitate to repeat the experiment; indeed, this is supported by our data showing that 35.7% of studies conducted an experiment only once. However, as demonstrated by Niepel *et al*. [18], the absence of biological replicates is a serious threat to the reproducibility of cell-based assays. They compared results from the same set of drug–response measurements performed at five laboratories and found that while various experimental and computational factors affected inter-laboratory reproducibility, biological factors (which varied in magnitude) were the most difficult to control. From this perspective, biological replicates should not be omitted from clonogenic assays.

Overall, 52.2% of the studies in our dataset reported a combined *n*_E_ and *n*_S_ of 1–3 or 3–1. This indicates that the authors of these studies were not aware of the distinction between biological and technical variance in clonogenic assay procedure. Since biological and technical variances are independent of each other, increasing the number of biological replicates does not compensate for reducing the number of biological replicates or *vice versa*. Biological and technical variance intrinsic to clonogenic assays should therefore be considered appropriately in future studies.

The present study demonstrates a huge variation in the dose rate used for clonogenic assays. The effect of dose rate on clonogenic survival is unclear, warranting further investigation to establish robust methods for inter-study comparison of clonogenic assay data obtained using different irradiation settings.

Clonogenic assay procedures intrinsically contain the sources of variation. Although the effect of the variations contained in each procedure on clonogenic survival has not been understood fully, it is highly important to at least record the experimental parameters used in the assays to ensure reproducibility. These parameters may include the following: cell line authentication, reagents used for cell culture, the timing of cell seeding (before or after irradiation), cell condition at irradiation (in monolayer or suspension), radiation source and energy, dose rate, the number of experiments, the number of samples per experiment and information on control treatment (e.g. vehicle reagent, empty vector or scramble siRNA).

In conclusion, we conducted a comprehensive literature review of 1672 papers and examined the standard of reporting of the methodologies used to perform *in vitro* radiosensitivity assessments using clonogenic assays. The data suggest that the reporting of the methodologies used for clonogenic assays in a considerable number of published studies is insufficient. Taken together, our data will raise awareness of the need for standardization and sound reporting of methodologies used to conduct clonogenic assays.

## Supplementary Material

Supplementary_Data_1_rraa064
